# Unique Symmetry-Breaking Phenomenon during the Self-assembly of Macroions Elucidated by Simulation

**DOI:** 10.1038/s41598-018-31533-z

**Published:** 2018-08-30

**Authors:** Zhuonan Liu, Tianbo Liu, Mesfin Tsige

**Affiliations:** Department of Polymer Science, University of Akron, Akron, OH 44325 USA

## Abstract

Various soluble hydrophilic macroions can self-assemble into hollow, spherical, monolayered supramolecular “blackberry”-type structures, despite their like-charged nature. However, how the 3-D symmetrical macroions prefer to form 2-D monolayers in bulk solution, especially for the highly symmetrical “Keplerate” polyoxometalates and functionalized C_60_ macroions has been a mystery. Through molecular dynamics simulations, using a model specifically designed for macroions in solution, the mechanism of this intriguing symmetry-breaking process is found to be related to the apparently asymmetric charge distribution on the surface of macroions in the equatorial belt area (the area which can be effectively involved in the counterion-mediated attraction). As a result, the electric field lines around macroions during the self-assembly process clearly show that the symmetry-breaking happens at the dimer level effectively defining the plane of the self-assembly. These findings are expected to contribute to our fundamental knowledge of complex solution systems that are found in many fields from materials science to biological phenomena.

## Introduction

**Figure 1:**
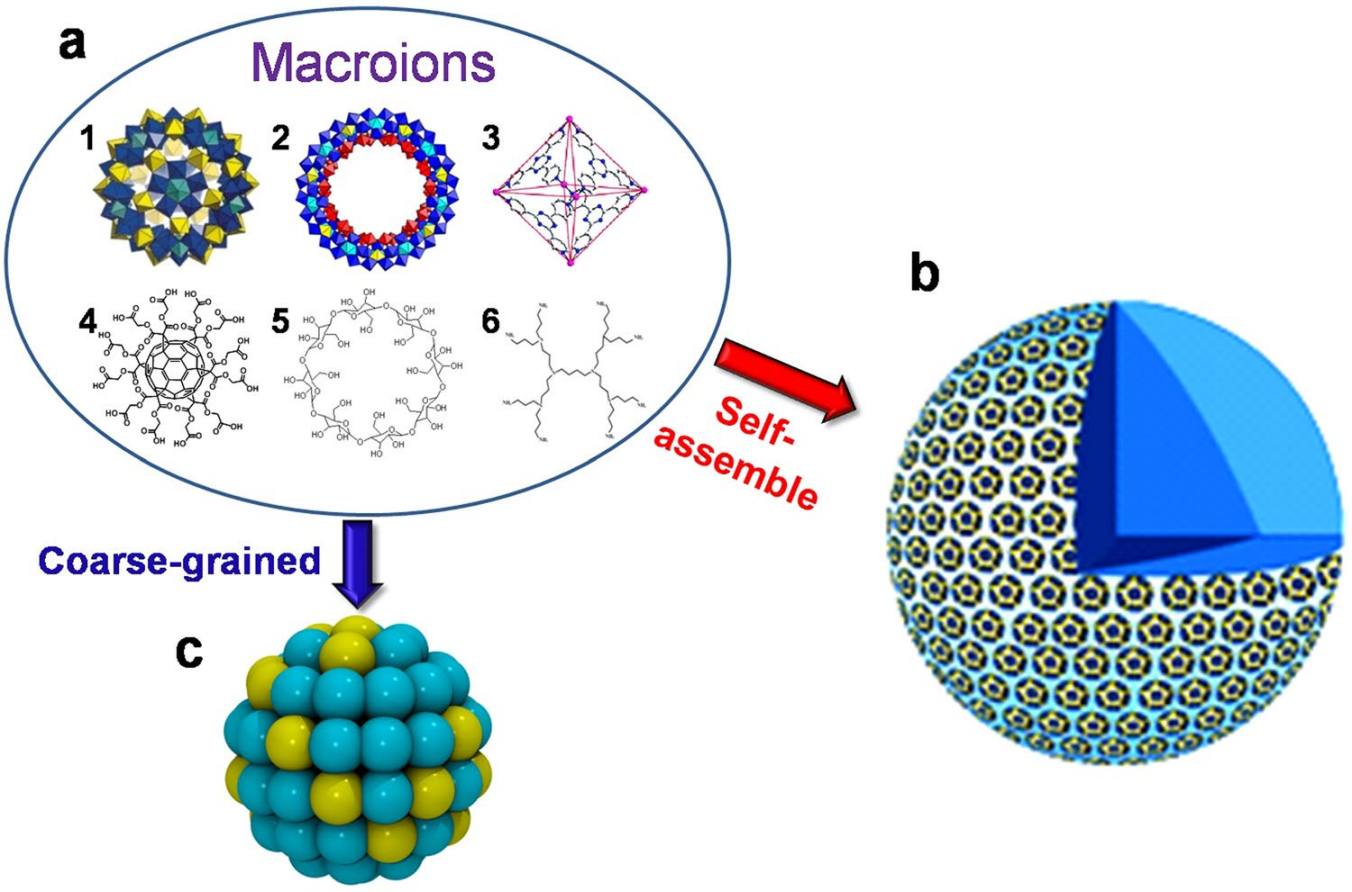
Coarse-graining of various macroions that form blackberry structures. (**a**) Examples of different kinds of macroions, including inorganic metal-oxide molecular clusters (1, 2)^8–13^, metal-organic nanocages (3)^18–21^, functionalized fullerenes (4)^15^, cyclodextrins (5)^14^ and dendrimers (6)^17^. (**b**) A typical blackberry structure self-assembled from metal-oxide molecular clusters (a1), which is a monolayer hollow sphere. (**c**) A coarse-grained model designed for general spherical macroions. In this model, the cyan beads have only VDW interactions while the yellow beads have both VDW and electrostatic interactions.

Hydrophilic macroions possess fascinating solution behaviors. Such large ions cannot be described by either Debye-Hückel theory^1^ for simple ions (due to their large sizes) or the DLVO theory^2,3^ for colloids because they still form thermodynamically stable solutions and the van der Waals (VDW) forces are very weak. Recent studies indicate that the like-charged macroions can strongly attract with each other when carrying moderate amount of charges, leading to the reversible formation of thermodynamically stable, hollow, spherical, and monolayered “blackberry”-type structures in polar solvents^4–7^. Vairous macroions (1-6-nm size) are found to do so, such as inorganic metal-oxide molecular clusters^8–13^, polyhedral oligomeric silsesquioxane^14^, functionalized fullerenes^15^, dendrimers^16,17^, metal-organic nanocages^18–21^, bio-macromolecules and small nanoparticles^22,23^ (Fig. 1). The blackberry structure formation has been confirmed to be due to the counterion-mediated attraction^4–7^. When the macroions have inhomogeneous surface charge distribution, tubular shaped assemblies have been observed^24^. Simulations have provided several important information in understanding the diffusion of macroions in solution and the distribution of water molecules and counterions around them^25–29^.

Our recent simulation study agrees with the hypothesis that the attraction between the like-charged macroions comes mainly from electrostatic interactions mediated by their counterions^25^. The VDW interaction is also contributing to the attractive forces, but we found the magnitude of this interaction to be about two orders of magnitude smaller than the electrostatic interaction for 2.5-nm-size spherical macroions^25^. Further investigations have discovered the chaotic nature of the electrostatic forces among macroions and their counterions, which dramatically decelerates the formation of self-assembled structures and makes the process more statistically dependent. The effect of macroionic charge density was also studied, which showed how the interactions between macroions as well as the dynamics of both macroions and counterions are dependent on the charge density^25–29^.

The most intriguing remaining question is why the macroions assemble into hollow, spherical structures? Many types of macroions, such as the Keplerates^30,31^, C_60_ and some nanocages, are structurally isotropic, which is different from the structurally-anisotropic surfactants. To form the hollow, spherical blackberry structure, the macroions need to have stronger intermolecular attraction along certain directions in a homogeneous bulk solution. That means, a symmetry-breaking process should take place, but how that happens is still a major mystery. In addition, the effect of macroionic size on their self-assembly behavior is also unclear.

## Results and Discussion

The effect of macroionic size on their self-assembly behavior has not been explored through simulation so far, to the best of our knowledge, probably due to the huge computational cost when trying to simulate macroions by all-atom molecular dynamics approaches. To overcome this difficulty, our coarse-grained model that has been used in a previous study^25^ is expanded to macroions with different sizes. The original CG model was designed to study the source of attraction between macroions in solution and the general self-assembly behaviors of various types of macroions, thus each macroion is represented by a hollow spherical structure which has both charged and uncharged beads on the surface, to mimic the structure of the well-studied 2.5-nm-size spherical “Keplerate” metal-oxide molecular cluster {Mo_72_Fe_30_}^30,31^. The counterions and solvents used with this model are also coarse-grained (the details are described in supporting information). Because of the simplicity and flexibility of this CG model, it can be easily expanded to macroions with different sizes and charge distributions, and simulations of larger macroions (up to 10 nm) using these types of CG models are accessible.

**Figure 2:**
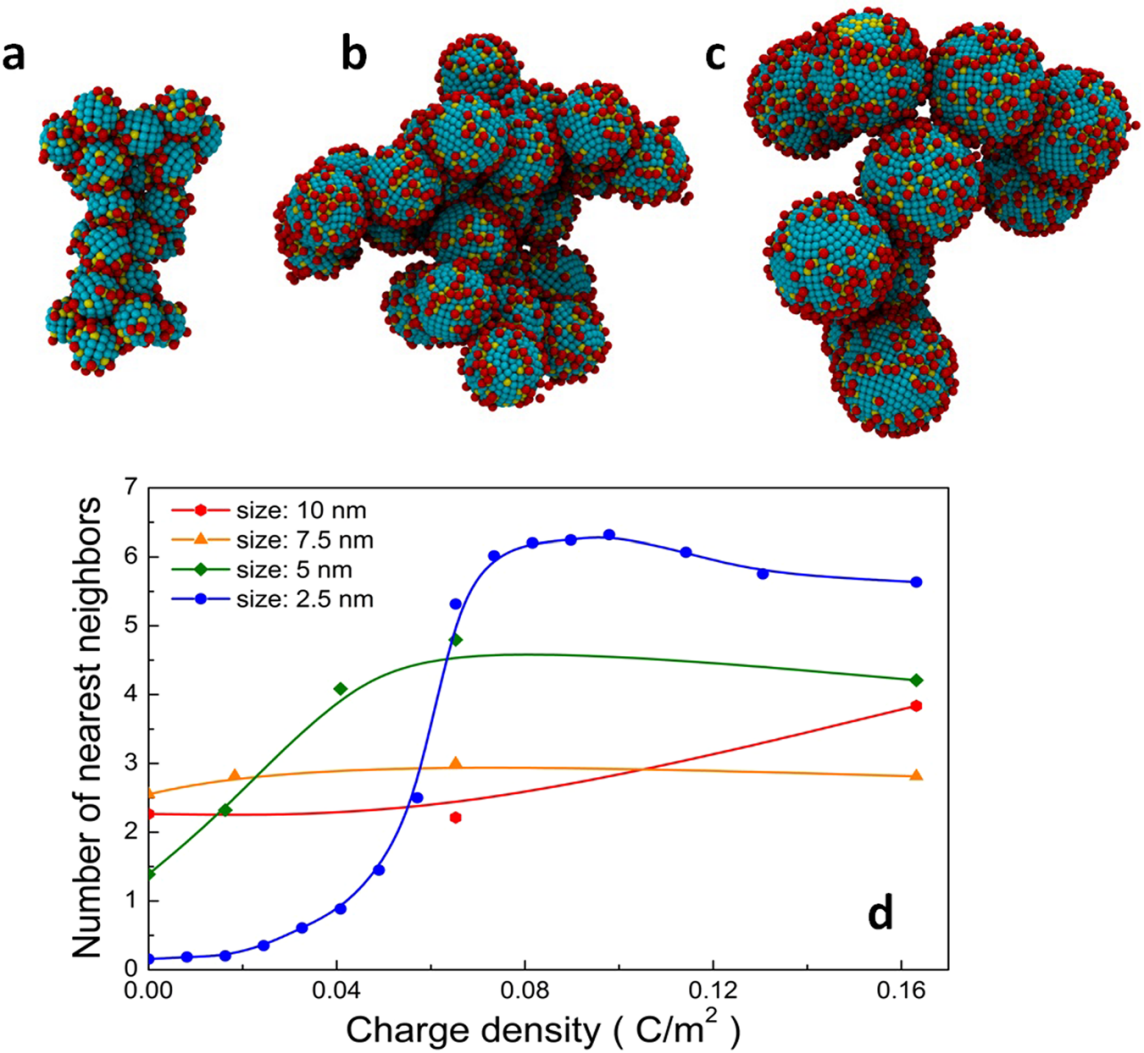
Size effect on the self-assembly behavior of macroionic solutions. (**a**–**c**) The final assembled states of macroions with a size of 2.5, 5.0 and 7.5 nm, respectively. The uncharged beads on macroions are cyan, while the charged ones are yellow. The counterions are red. Explicit solvent molecules are hidden for clarity. (**d**) The number of nearest neighbors for macroions with size ranging from 2.5 to 10 nm and charge density ranging from 0 to 0.16 C/m^2^.

In order to study the effect of the macroionic size on their self-assembly behaviors, CG models of macroions with four different sizes from 2.5–10 nm were created (Fig. S1), and four systems were built accordingly. All species were initially randomly distributed in solution, followed by equilibration for >200 ns. The visualization of simulation results is shown in Fig. 2a–c. Regardless of the size difference, each of the four types of macroions forms into one large aggregate, indicating that either electrostatic or VDW forces drives the self-assembly process.

Further investigation was performed by varying the charge density (0–0.16 C/cm^2^) on each type of macroion, in order to understand the role of the electrostatic interaction in the assembly process. Interestingly, for macroions >7.5 nm in size with low charge densities, a great tendency to aggregate was found. The macroion-macroion radial distribution function (RDF) was then calculated for each system (an example is shown in Fig. S2 with detailed discussion). The number of nearest neighbors is then calculated by integrating the area under the first two peaks observed in the RDF calculations, leading to a full-spectrum comparison of the charge density and size effect on the self-assembly of macroions (Fig. 2d). Several intriguing features are observed from this comparison: first, as the size of the uncharged macromolecules increases, larger assemblies tend to form, indicating that the strength of the VDW interactions between macroions correlate with their sizes; secondly, increasing the charge density of macroions always has a positive effect on the tendency of forming assemblies, implying that in all cases the electrostatic forces are attractive. However, as the macroionic size increases, the electrostatic attraction between macroions diminishes, and may even become repulsive as predicted by the DLVO theory of large colloids. This observation supports the experimental results that the electrostatic interaction is responsible for the blackberry structure formation of macroions, and is dominant when the macroionic size is less than ~10 nm. VDW attractions start to dominate and cause electrostatic forces to become less attractive and even repulsive as the macroions grow larger, leading to typical colloidal behavior such as precipitation from the solution.

The most interesting yet unsolved mystery is the hollow, spherical, single-layered morphology of the blackberry structure. An attempt has been made to observe blackberry structure formation using our CG model for macroions in which their size, surface charge density, and charge distribution can be easily altered to simulate a full range of VDW/electrostatic interaction between them. Although it is difficult to simulate the formation of a whole blackberry structure, because it may contain thousands of single macroions; it is possible to simulate the early stage of the formation since the macroions should initially self-assemble into a 2D monolayer. However, simulation of a large system containing 50 macroions (each with 2.5-nm-diameter and 20- charges), 1,000 counterions, and 5 million solvent molecules didn’t show any sign of 2D monolayer formation, only a large 3D irregular aggregate was formed. This result is reasonable because there should not be directional preference for the assembly since the macroions were assigned random charge distribution on their surface. Then the key question is: how do macroions initially self-assemble into a monolayer structure? Since many macroions have rigid structures, in order to break the isotropic symmetry and form a monolayer, the positions of the charged sites on the macroions may be reconfigured depending on the solution environment. Thus we hypothesize that the charge distribution on macroions is the key for the formation of blackberry structure.

**Figure 3:**
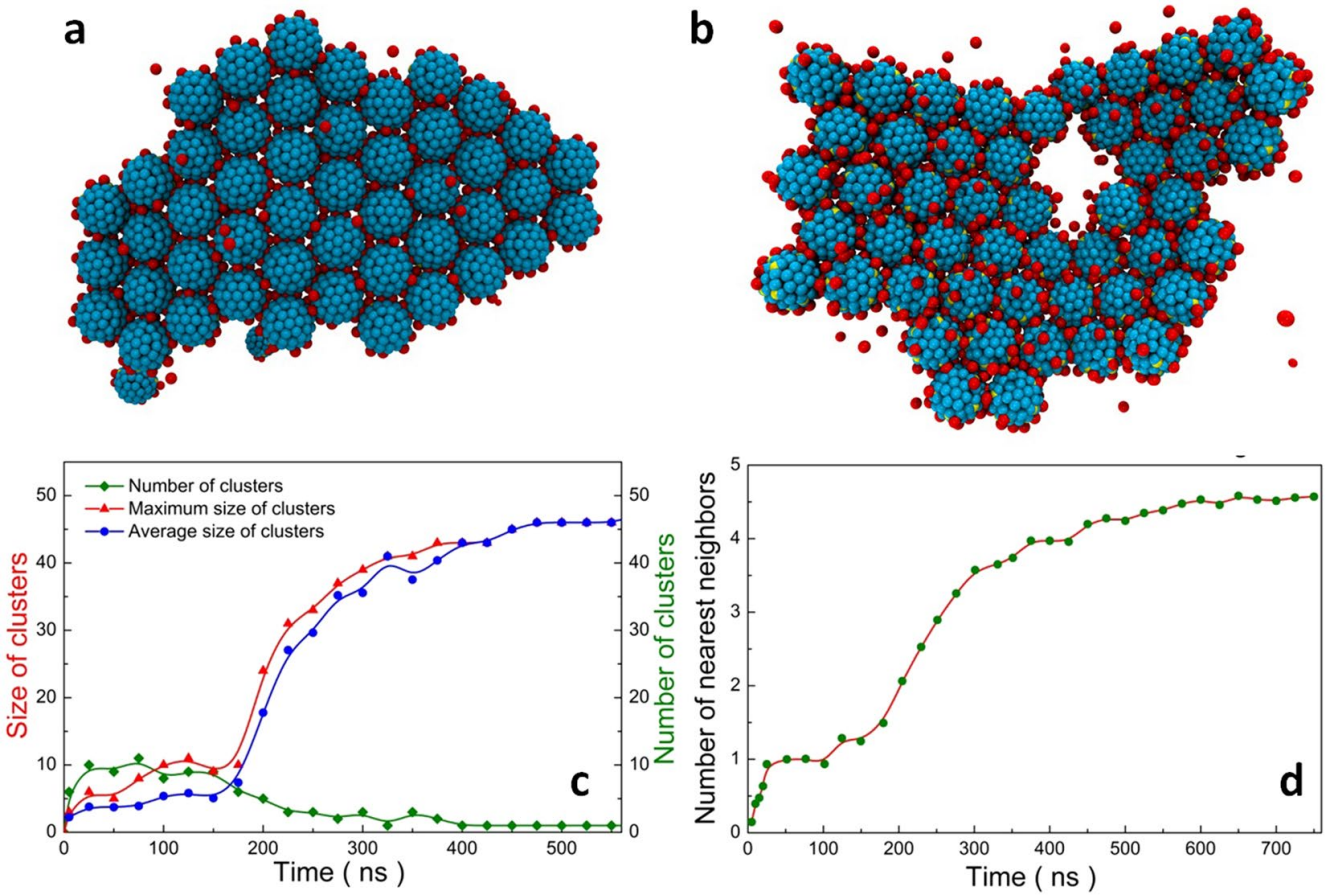
2D monolayer structures formed by macroions with equatorial charge distributions and the time evolution. (**a**) Final assembly of macroions with an equatorial charge distribution (10 charges). (**b**) Final assembly of macroions with 20 charges distributed on the equator and two “tropics”. Solvents are hidden for clarity. (**c**)The size and number of assembled clusters plotted as a function of time. (**d**) The average number of nearest neighbors of macroions plotted with time. The solid lines are drawn to guide the eyes.

To verify our hypothesis, various types of macroions covering a big range of charge densities and charge distributions were created. Figure S3a–h show eight types of representative macroions which have moderate charge densities and different charge distributions. Interestingly, among macroions with different charge distributions, some did self-assemble into nicely packed 2D monolayers, when the charges are distributed near the “equator” of the macroions (Fig. S3d–f) that they can form 2D monolayers. The others all ended up forming 3D aggregates. Careful examination of the monolayers (Fig. 3a,b) reveals that the macroions seem to have a well-defined hexagonal packing (also confirmed by the RDF characterizations, Fig. S4), which was also observed in {Mo_154_} solution although the packing was only short-ranged^12^. Furthermore, the monolayers in solution seem to be not rigid most of the time, instead they demonstrate fluctuating surfaces. Some defects are also observed (Fig. 3b).

Since the charge distribution is crucial for the 2D monolayer formation, in reality would the charges on macroionic surface redistribute during the self-assembly process? If they do, could the reason be that the new states with redistributed charges are more energetically favorable? Several batches of simulations were then performed. In each batch, different macroionic solution systems were built with same conditions except the charge distributions on the macroionic surface (Fig. S5). All comparisons show that the systems with macroions having charges distributed close to their “equators” always have the lowest total energy after forming stable assembled structures. These results indicate that if the charges on the macroions are movable, they may eventually redistribute closer to the macroion’s equatorial area to lower the system energy, resulting in a 2D monolayer structure that slowly forms over time.

Furthermore, the dynamic process of 2D monolayer formation was evaluated. Figure S6a–f show a typical monolayer formation from macroions carrying 10 charges on the “equator”. The self-assembly process is very slow as can be seen in the figure, fully consistent with experimental observations^13^. In addition, different methods were developed to quantify this process. Figure 3c shows how the average size and number of the oligomers formed by macroions change with time. A clear sigmoidal curvature is observed, again consistent with our previous observations^32^. Moreover, the calculated number of nearest neighbors (Fig. 3d) also displays a similar sigmoidal feature. Accordingly, an interesting picture is revealed: the slow initial induction comes from the difficult oligomer formation, possibly due to the short-ranged and chaotic nature of the electrostatic attraction^25^ mediated by counterions. As larger oligomers slowly form, the increase in the attractive electrostatic forces accelerates the assembling process; eventually the dramatic drop in local concentration of free macroions and oligomers results in very slow completion of large monolayers.

**Figure 4:**
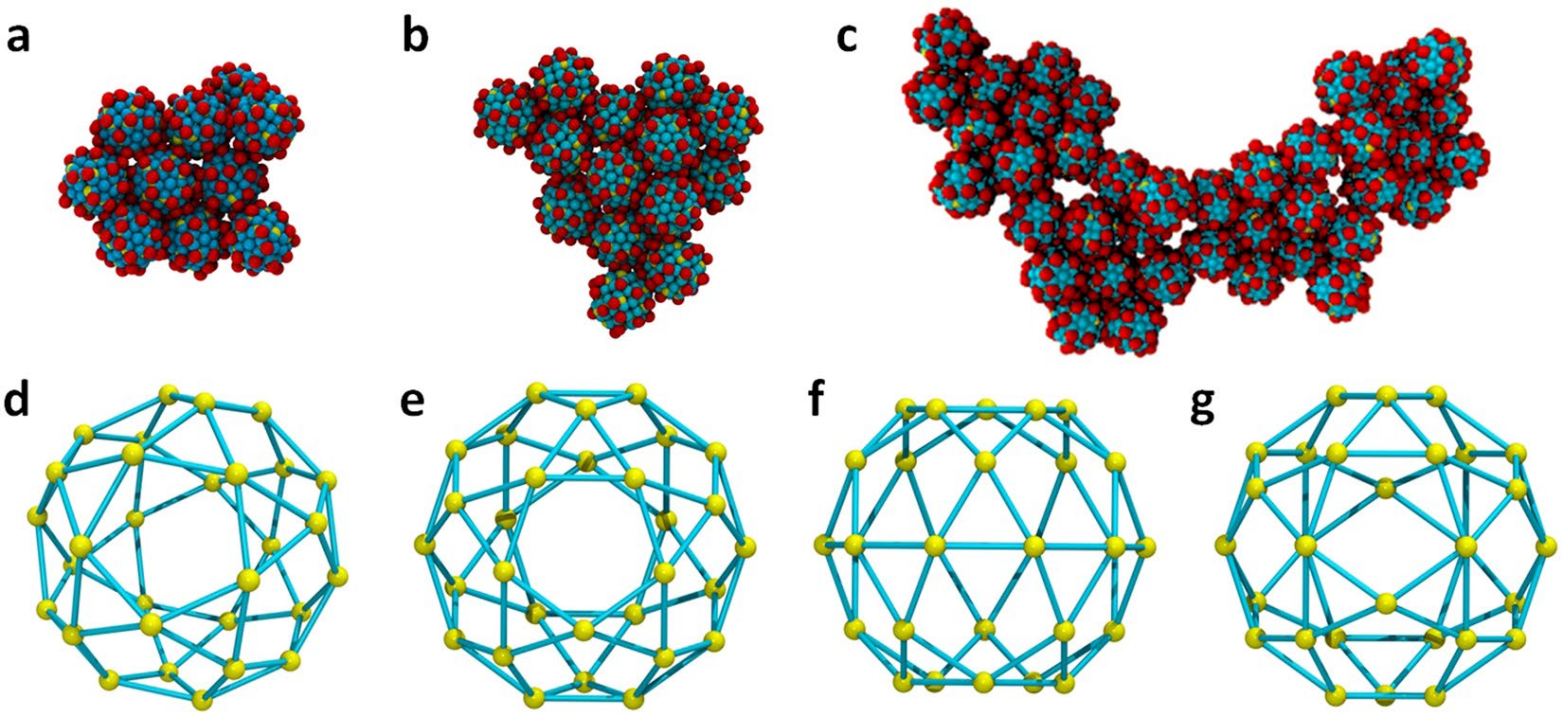
Self-assembly of macroions with a quasi-isotropic (icosidodecahedron shaped) charge distribution. (**a**) Self-assembled structure of 10 macroions. (**b**) After adding four macroions one by one into a. (**c**) 2D monolayer merged from four small monolayers as shown in a. (**d**) The shape of an icosidodecahedron. (**e**) The top view of this polyhedron when sitting on one of its pentagons on the surface. (**f**) The side view when sitting on one of the pentagons. (**g**) The side view when sitting on one of the triangles.

A very critical question is why the structurally isotropic macroions such as Keplerates, with apparently isotropic charge distributions, also self-assemble into 2D monolayers prior to the blackberry structure formation. To better understand this, a model was designed: 30 negative charges were symmetrically assigned on the 30 vertices of an icosidodecahedron on the surface of a macroion (as shown in Fig. S7a–c), identical to the locations of possible charge sites on {Mo_72_Fe_30_} surface. Surprisingly, such macroions also forms a 2D monolayer (Fig. 4a). Although a minor defect is observed, the macroions pack in hexagonal closest-packed structure, like the ones with charges distributed close to their equators. Compared with other macroions having purely isotropic charge distributions that we have studied before, such as the ones with charges distributed on the vertices of a cube (Fig. S3c) or the ones with all surface beads charged^25^, this result seems particularly intriguing since all the others form 3D aggregations instead of 2D monolayers.

To better understand the reason, we carefully examined the symmetry of charge distribution on the icosidodecahedron. Figure 4e,f show the top and side views of this structure, when it sits on one of its pentagons on the surface. No matter how one rotates the sphere, the side view of the vertices shows an anisotropic charge distribution: the density of the vertices on the “belt” surrounding the sphere seems to be relatively higher. Since the average distance between the surface of macroions after forming a monolayer is ~0.6 nm (Fig. S4 and experimentally confirmed) and assuming the counterions need to stay in the region between two macroions where the distance between the surfaces of the macroions is ~1.0 nm in order to effectively mediate the attraction (see Fig. S7d), we can then define a belt area which has a width of ~1.4 nm on the surface of macroions. When placing a macroion on one of its pentagons as shown in Fig. 4f (see also the top view in Fig. 4e), the charge density inside this belt area is about 57% higher than the rest of the surface area. This explains the mechanism of the monolayer formation: the higher charge density around the belt area breaks the symmetry and attracts more counterions to this area and becomes the plane for a 2D self-assembly, similar to the case of equatorially charged macroions like {Mo_154_}. However, when placing the macroion on one of its triangles, the charge density inside this belt area is about 47% lower than the rest of the surface area, which explains why most of the macroions in the monolayer align themselves along their pentagons instead of the triangles. In general, macroions with even very slight anisotropy in their charge distribution may cause a “belt” area with higher charge density to form around them, which enable them to form a 2D monolayer structure. This means, in our hypothesis regarding the relationship between the charge distribution and the ability to form blackberry structures, the charges on the macroions need not move very far, even a slight tendency of redistributing the charges closer to the equator may lead to the formation of 2D monolayer structures.

Two approaches of expanding the size of the monolayer structure were designed to test if the monolayer structure was formed out of coincidence. The first one is to introduce the same type of macroions one at a time with corresponding counterions and solvent molecules into the solution which contains the monolayer. As expected, the single macroions slowly merged with the monolayer on the edge, leading to an expansion of the packing (Fig. 4b). Another approach is to replicate the small monolayer four times and mix them in a large solution system. Again the monolayers quickly merged with each other along their edges and formed one large monolayer (Fig. 4c). Comparing the two tests, the merging of the monolayer and single macroions is much slower but leads to a fine packing structure, while the merging of monolayers is faster due to the stronger electrostatic interactions, but the assembled structure is less perfect (some defects can be observed in Fig. 4c). These observations also correlate with the sigmoidal curvature in the self-assembly process, and maybe related to the size difference of blackberry structures formed by different types of macroions.

**Figure 5:**
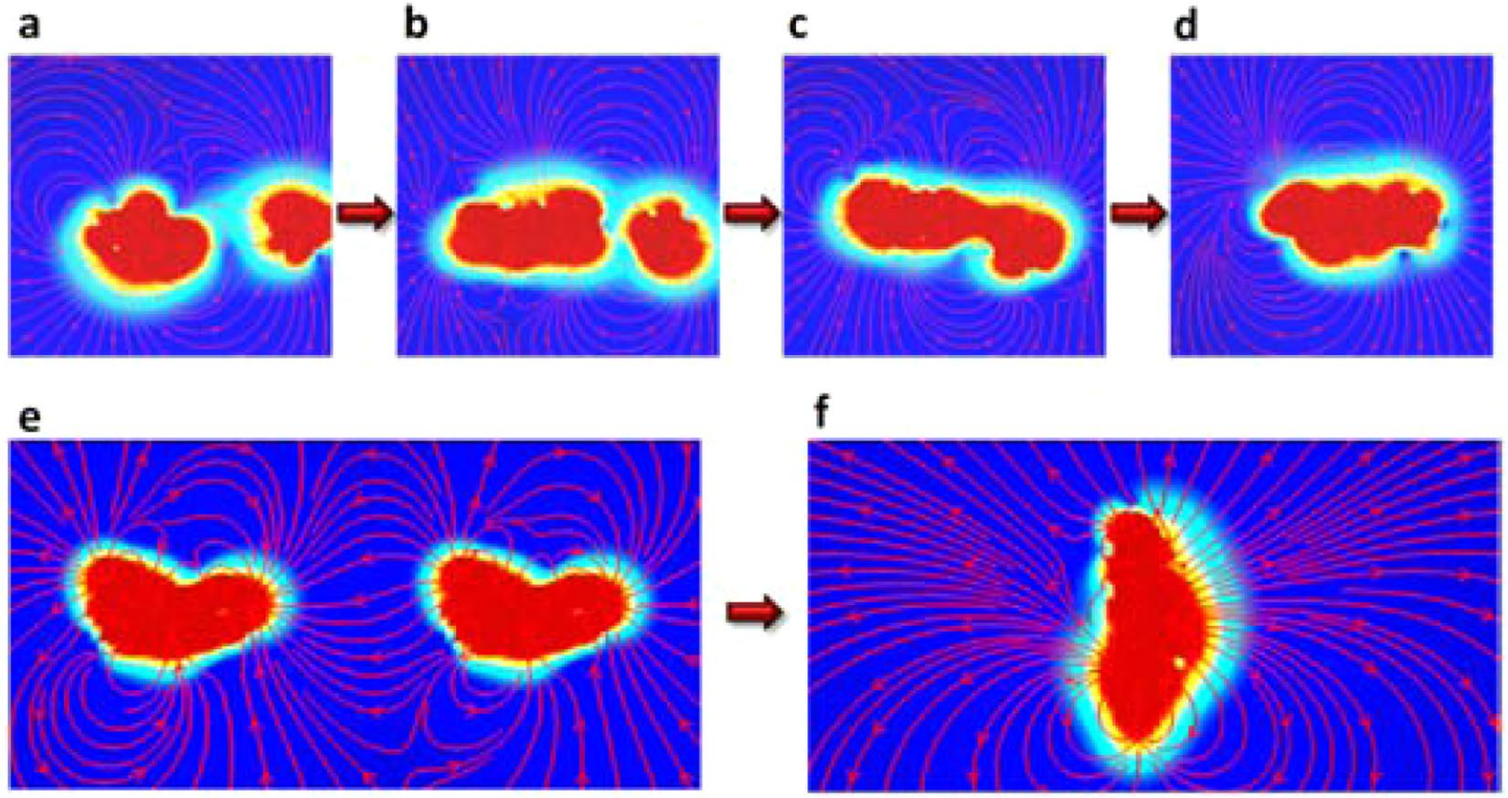
Electric field characterization during the self-assembly process of macroions with a quasi-isotropic charge distribution. (**a**–**d**) The evolution of the electric field in the system where a single macroion merges with a monolayer (as shown in Fig. 4a). (**e**,**f**) The initial and end states of two monolayers merging with each other. The coloring method is the same as in Fig. S8.

To better understand why the monolayers grow along the edges instead of stacking on top of each other or crossing each other’s planes, and how the single macroions gradually merge with monolayers, an approach of characterizing the electric field surrounding the charged species was developed to visualize the electrostatic interaction among them, since it has been proved that the electrostatic force is the driving force for the self-assembly^25^. A test of two single macroions using this method reveals how counterions mediate the attraction (Fig. S8). Once a dimer is formed, a resultant electric field that is attractive to other macroions is observed around the “belt” area of the dimer that dictates the plane of monolayer growth direction. Further investigation illustrates how the single macroions merge with a monolayer (Fig. 5a–d, a more detailed process is shown in Fig. S9), and how two monolayers merge with each other (Fig. 5e,f). The self-assembly process becomes clear now. A resultant electric field that is attractive to other macroions is clearly observed around the plane of the monolayer (Fig. 5). This electric field is responsible for the monolayer to grow larger by attracting other macroions and we hypothesize that this will finally result in the formation of the blackberry structure.

In conclusion, we have performed large-scale CG molecular dynamics simulations to address the fundamental question that how the symmetry-breaking process is achieved during the blackberry structure formation in dilute solution. We believe our general approach of understanding the process of self-assembly of charged molecules in solution will open a new direction in the study of the self-assembly and the nature of interactions in the broadly defined macroionic solutions, which cover a variety of fields from materials science to biological phenomena.

## Methods

In order to study the general self-assembly behaviors of various hydrophilic macroions, a versatile coarse-grained (CG) model that represents macroions of varying charge density and size was developed in a previous work^25^ and applied in this work. The design of the CG model is based on the molecular structure of typical macroions such as polyoxometalate molecules. One macroion is represented by one hollow sphere with two different types of beads on the surface. The surface beads are either uncharged or charged in order to represent the van der Waals and electrostatic interactions among macroions, counterions and solvent molecules in the solution. The size and charge values of each surface bead, the size of the macroion, and the number of charged beads and their distribution on the surface can all be tuned to represent a specific type of macroion. The surface beads of a macroion are designed to move as one rigid body, which benefits the efficiency of the molecular dynamics simulations since the intra-molecular interactions within each macroion are not considered. This design is based on the assumption that the shape, size and composition of each macroion will not change in the process of self-assembly in solution, which means that there is no obvious relative movement of atom groups on the surface of each macroion.

The Lennard-Jones (LJ) 12-6 potential energy function was used to describe the van der Waals interactions between different kinds of species in the solution:

$${{U}}_{{LJ}}({r})=\{\begin{array}{ll}4{\varepsilon }[{(\frac{{\sigma }}{{r}})}^{12}-{(\frac{{\sigma }}{{r}})}^{6}], & {r}\le {{r}}_{{c}}\\ 0, & {r} > {{r}}_{{c}}\end{array}$$

Here U_LJ_(r) is the VDW interaction between pairs of beads separated by a distance of r; ε is the energy term and r_c_ is the cut-off distance for LJ potential. The CG force field parameters for solvent were taken from the model of water in MARTINI force field. In this CG model of water, one bead is equivalent to four water molecules. The CG beads on the surface of macroions also have the same size (5 Å) and van der Waals interaction parameters as the solvent beads to account for their hydrophilic characteristic, so are the counterions. The CG force field parameters of the solvent can be tuned to represent a good or bad solvent for the macroions.

In this work all coarse-grained molecular simulations were performed by LAMMPS package, and the force field parameters used in all CG simulations are unitless quantities (LJ-style units). Without losing generality, LAMMPS has set the fundamental quantities, such as mass, sigma, epsilon and the Boltzmann constant to 1, and the specified masses, distances and energies in simulations are multiples of these fundamental values. The formulas that correlate the reduced (unitless) quantities to the same quantities with real units is provided in the LAMMPS manual^33^. Therefore one can use the mass, sigma and epsilon values for a specific material and convert the simulation results from a unitless LJ-type simulation into physical quantities. The coarse-grained simulation studies performed in this work all used reduced units, while when interpreting the force field parameters in the discussions, equivalent real units are normally used in order to depict a more realistic picture. Table S1 in the SI shows detailed conversion between the reduced LJ-style quantities and real quantities. The conversions have been carefully performed according to the LAMMPS manual^33^. In the CG model of macroionic solutions the ε of all pair interactions between all kinds of species is set to 4.5 kJ/mol, and the σ is set to 5 Å in order to obtain a good solvent environment. The cut-off distance r_c_ is set to 12.5 Å for all LJ interactions.

Furthermore, the interactions between the charged beads on the surface of the macroions and the corresponding counterions in the solution were described by the Coulomb pair-potential:

$${{\rm{U}}}_{{\rm{C}}{\rm{o}}{\rm{u}}{\rm{l}}}={{\rm{k}}}_{{\rm{q}}}\frac{{{\rm{q}}}_{{\rm{\alpha }}}{{\rm{q}}}_{{\rm{\beta }}}}{{\rm{r}}}$$

Here U_Coul_ is the Coulomb potential for a pair of beads separated by a distance of r, q_α_ and q_β_ are the charges on each bead, respectively; and k_q_ = 1/4πε_0_, where ε_0_ is the permittivity of vacuum. Long range Coulombic interactions were calculated using the particle-particle/particle-mesh (PPPM) Ewald algorithm^34^. In the current model, each charged bead on the surface of macroions has one negative charge, and accordingly each counterion has one positive charge, while the solvent beads are not charged.

All the beads on each macroion move as one rigid body, thus the optional RIGID package in LAMMPS was installed and utilized for this purpose. The time step used in these coarse-grained MD simulations is 0.005 in reduced CG unit, equivalent to 10 fs. Each simulation was started with distributing the macroions, counterions and solvent molecules randomly in the system. The code written for distributing different types of particles in the initial simulation box ensures that no solvent or counterion particle is in the cavity of a macroion. All simulations was performed under isothermal-isobaric (NPT) ensemble in the beginning at a temperature of 1.0 and a pressure of 0.1 in reduced unit, and once the density of the system reaches a stable value, new simulations were continued from the restart configurations with isothermal (NVT) ensemble to enhance the computational efficiency. Due to the slow dynamics of macroions, most of the simulations took more than 100 ns to reach a state where the assembled structures are stable.

### Statistical information

To ensure the various morphologies generated from the MD simulations are in the equilibrium state, same simulations have been repeated several times from different initial distributions, and all tests show that the assembled structures are similar in equilibrium state.

## Electronic supplementary material

Supplementary Information

## Data Availability

All relevant data are available from the authors upon reasonable request.
