# Supplementary material for: Unique Symmetry-Breaking Phenomenon during the Self-assembly of Macroions Elucidated by Simulation
Source: Sci Rep. 2018 Aug 30;8:13076. doi: 10.1038/s41598-018-31533-z (PMC6117333; doi:10.1038/s41598-018-31533-z)
Supplement: Supplementary file 1 — Supplementary Information [file 41598_2018_31533_MOESM1_ESM.docx]

**SUPPORTING INFORMATION**

Unique Symmetry-Breaking Phenomenon during the Self-assembly of Macroions Elucidated by Simulation

**Zhuonan Liu^†^, Tianbo Liu^†‡^, Mesfin Tsige^†‡^**

**^†^**Department of Polymer Science, University of Akron, Akron, OH 44325, USA.

**^‡^**Corresponding author. E-mail: mtsige@uakron.edu (M.T.) and tliu@uakron.edu (T.L).

**Simulation Methods**

In order to study the general self-assembly behaviors of various hydrophilic macroions, a versatile coarse-grained (CG) model that represents macroions of varying charge and size was developed in a previous work^1^ and applied in this work.

The coarse-graining simulation studies performed in this work all used reduced units, while when interpreting the force field parameters in the discussions, equivalent real units are normally used in order to depict a more realistic picture. Table S1 shows detailed conversion between the reduced LJ-style quantities and real quantities.

Table S1. The relations between reduced unitless quantities and real quantities used in the CG model of macroionic solutions.

|  | Reduced quantities | Real quantities |
| --- | --- | --- |
| Energy (*ε*) | 1.0 | 4.5 kJ/mol |
| Distance (*σ*) | 1.0 | 0.5 nm |
| Mass | 1.0 | 72 g/mol |
| Temperature | 1.0 | 540 K |
| Pressure | 1.0 | 59.8 mPa |
| Time | 1.0 | 2 ps |

**Supporting figures**

**
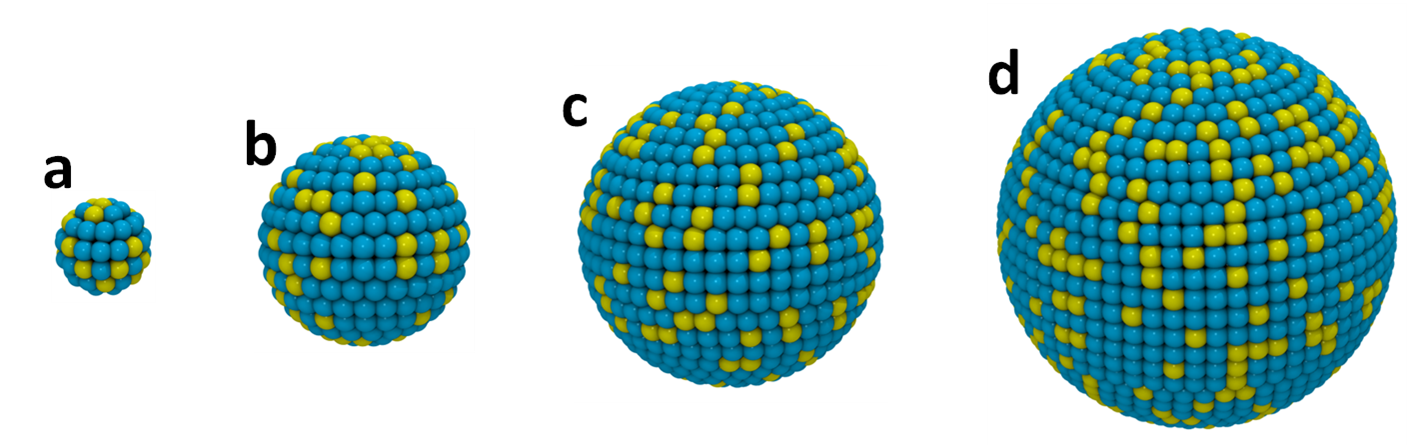
**

Figure S1. Coarse-grained models of macroions with different sizes and charge distribution. (a, b, c, d) CG models of macroions which have a size of 2.5, 5.0, 7.5 and 10.0 nm, respectively. The beads of blue color are the uncharged ones with VDW interactions only, while the yellow ones carry 1 negative charge each. The charges are randomly distributed on the surface of the macroions.

**
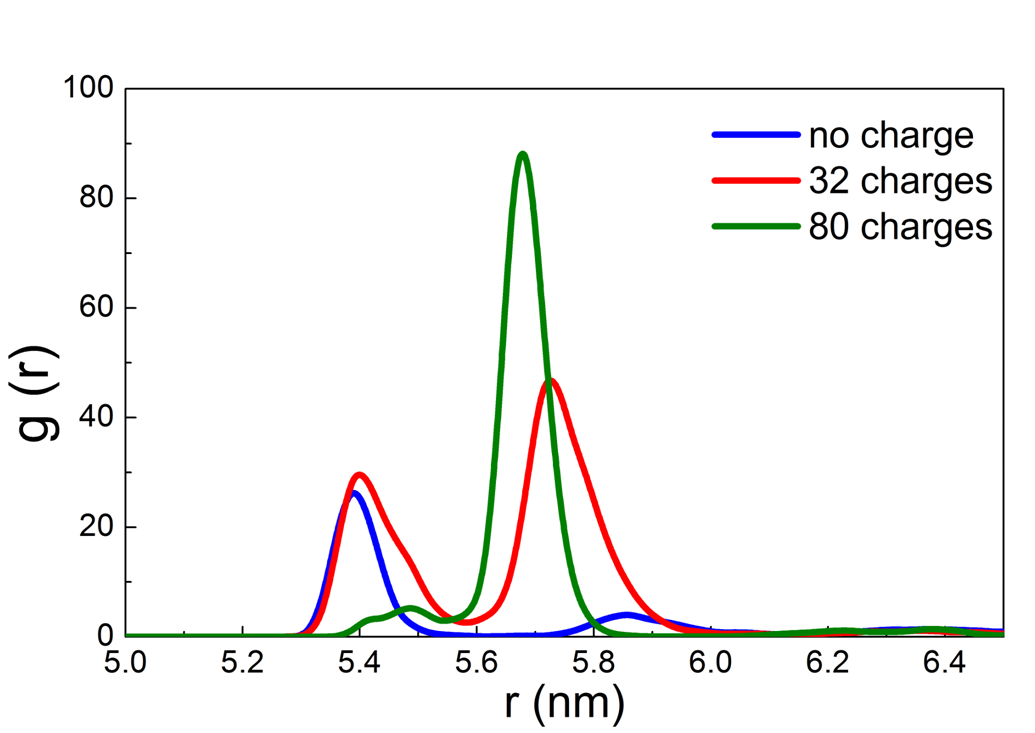
**

Figure S2. Radial distribution functions (RDF) of macroion-macroion pair with different charge densities. In all the three systems the macroions have the same size (5.0 nm) but different charge densities. The number of charges that each macroion carries is 0, 32 and 80 in three macroionic solution systems, respectively. This comparison of RDF calculations indicates that increasing the charge density on this type of macroion can enhance their ability to aggregate. The disappearance of the first peak around 5.4 nm for macroions with 80 charges indicates that the counterions are in between macroions to mediate the electrostatic attraction.

**
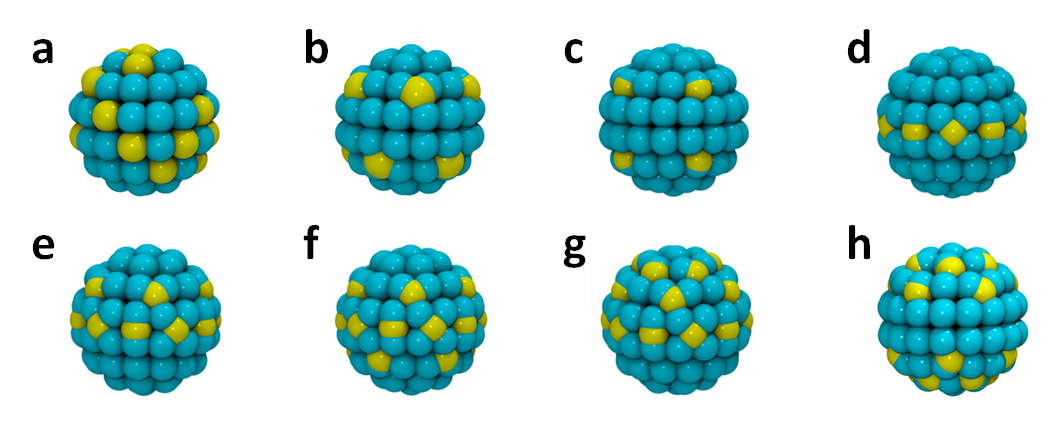
**

Figure S3. CG models of macroions with various charge distributions. The charge distributions are: (a) 20 charges randomly distributed on the surface; (b) 10 charges on the “tropics” (analogous to a globe); (c) 8 charges on the vertices of a cube (body diagonal 2.5 nm); (d) 10 charges on the “equator”; (e) 15 charges on the equator and one tropic; (f) 20 charges on the equator and both tropics; (g) 20 charges on half sphere. (h) 20 charges on top and bottom.

**
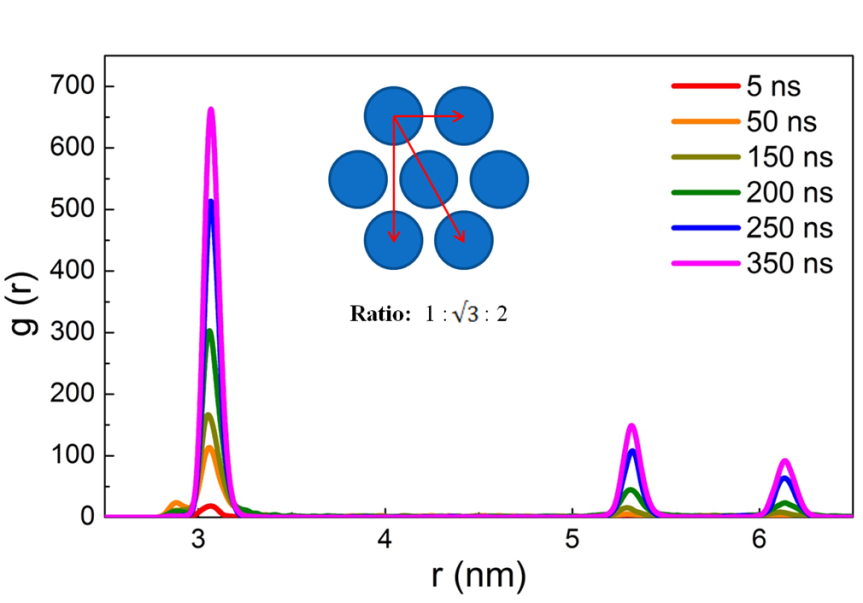
**

Figure S4. Radial distribution functions of macroion-macroion pair during the formation of a 2D monolayer. The results are calculated at different stages of the self-assembly process, shown in individual colors. After 350ns MD simulation the assembled structure barely changes with time.

**
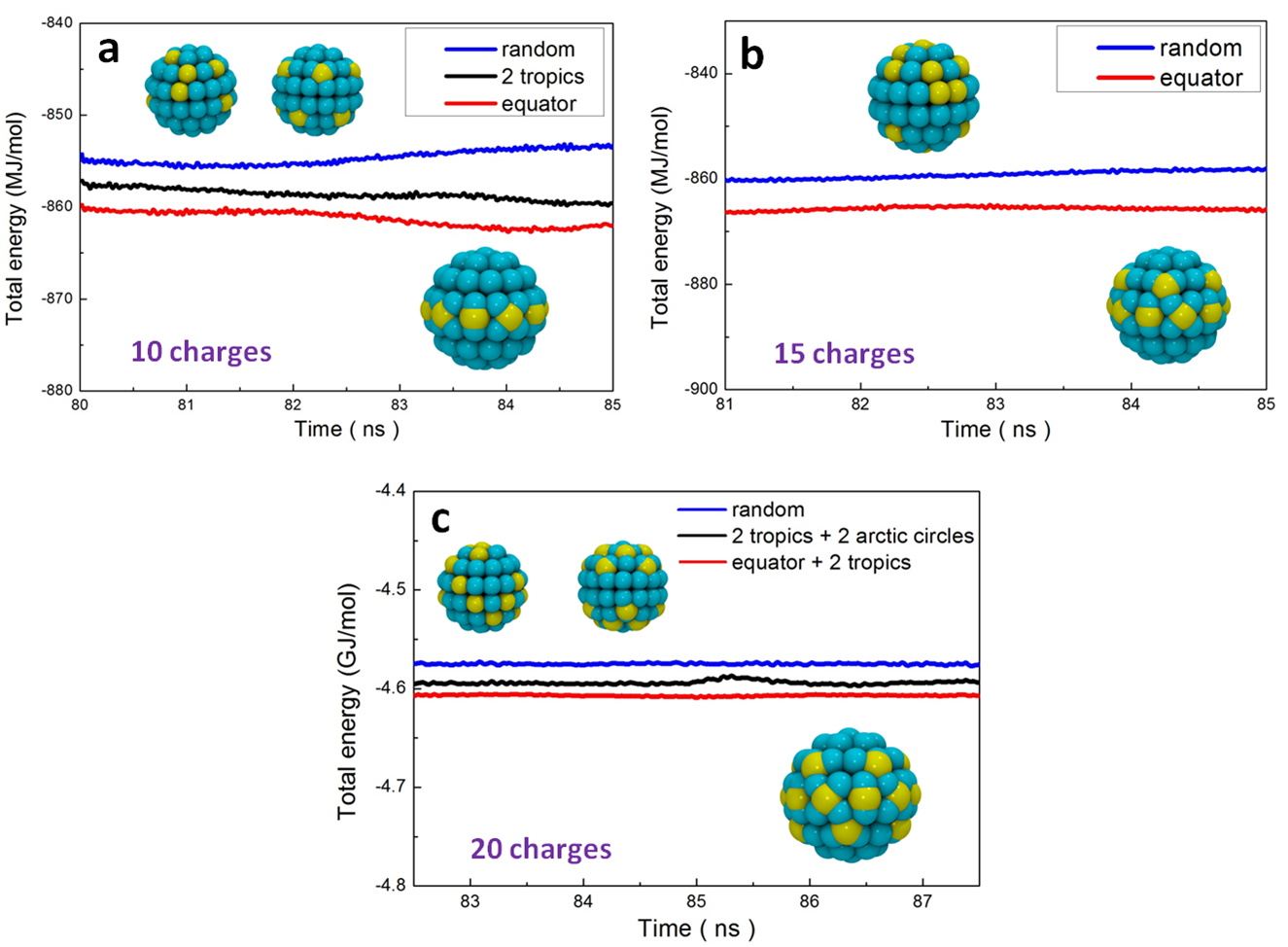
**

Figure S5. Comparisons of the total energies of macroionic solution systems with same charge density but different charge distributions. In each comparison, the number of macroions, counterions and solvents, and the charge density of the macroions in each system are set to the same value. (a) Three systems in which the macroions all have 10 charges on their surface but different charge distributions: random, tropical, and equatorial. (b) Similar comparison as a. The macroions all have 15 charges and the different charge distributions are random and a hybrid of equatorial and tropical. (c) Similar comparison as in a and b. All macroions have 20 charges and the results are after equilibrating the systems for more than 80 ns.

**
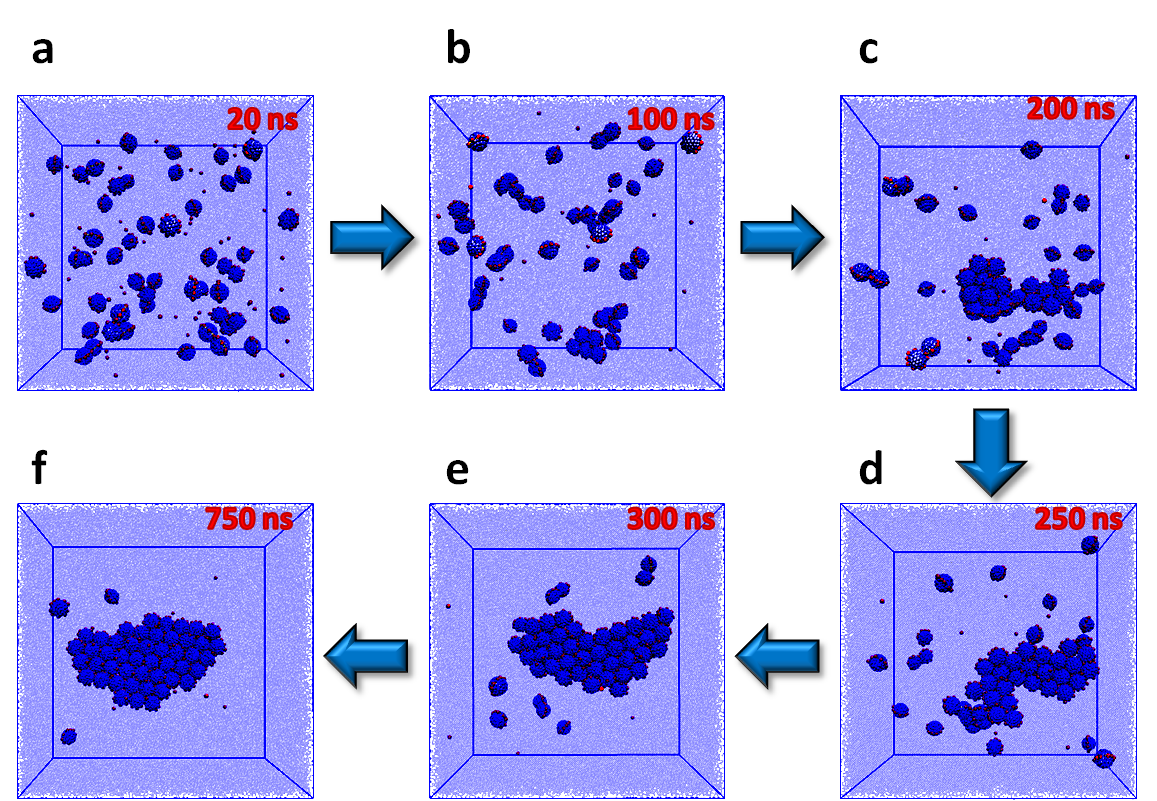
**

Figure S6. Time evolution of the self-assembling process of 2D monolayers. (a to f) Snapshots taken at 20, 100, 200, 250, 300, and 750 ns of a simulation in which 50 macroions (each carrying 10 charges on the “equator”) self-assemble into a 2D monolayer. The macroions are colored in deep blue and the counterions are red. The solvent molecules are also shown here in light blue color.

**
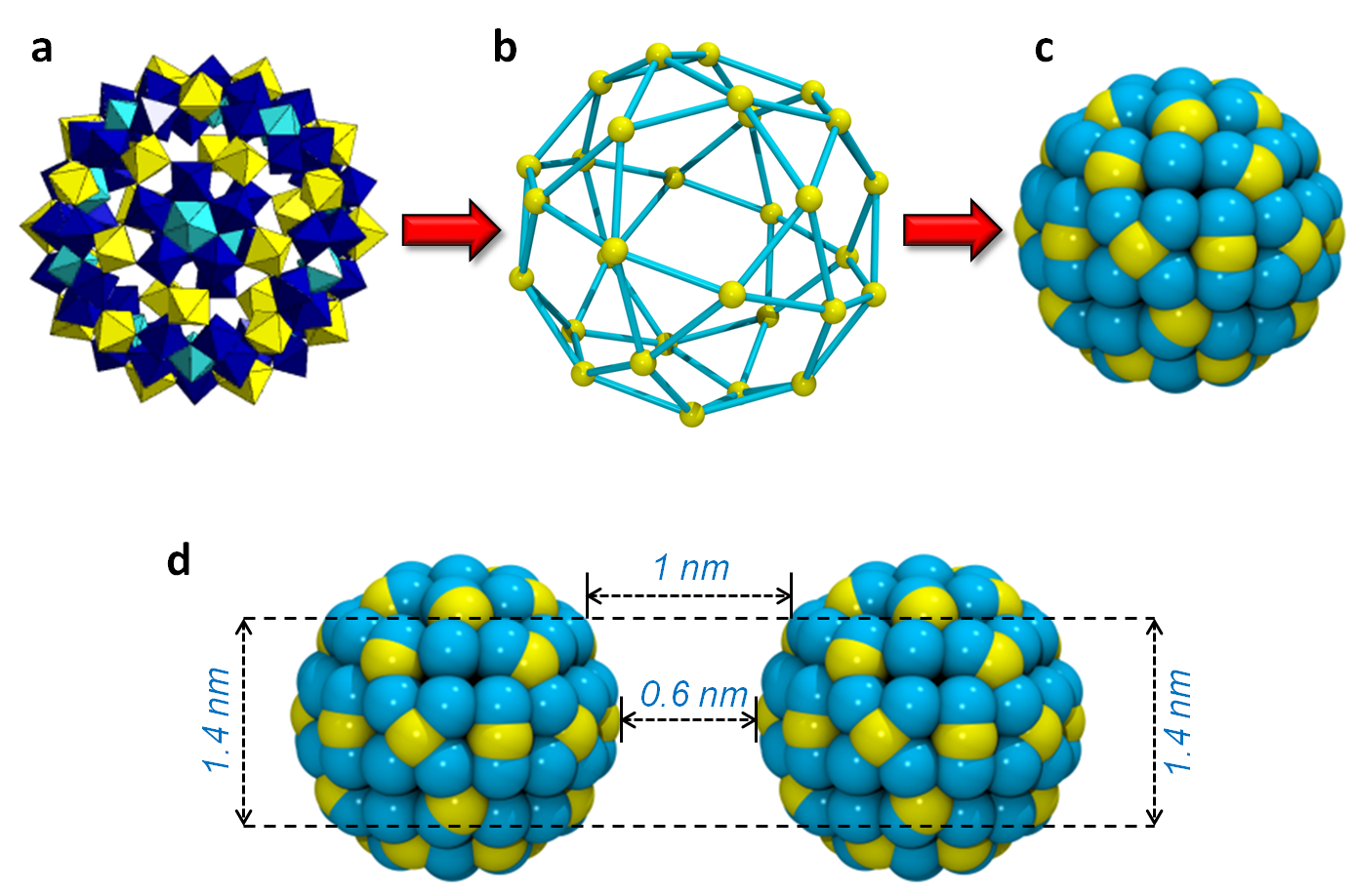
**

Figure S7. Coarse-graining of a macroion carrying 30 charges with an icosidodecahedron-shaped charge distribution. (a) The molecular structure of polyoxometalate molecule {Mo_72_Fe_30_}. (b) The appearance of an icosidodecahedron. (c) The CG model of macroions having the same icosidodecahedron-shaped charge distribution as {Mo_72_Fe_30_}. (d) The effective belt area on the surface of macroions. Since the average distance between the macroions on a monolayer assembly is about 0.6 nm, assuming the distance between the two surfaces of neighboring macroions for the counterions to effectively mediate the attraction is about 1 nm, then the belt area that may contribute to the attraction is about 1.4 nm in height on the spherical surfaces.

**
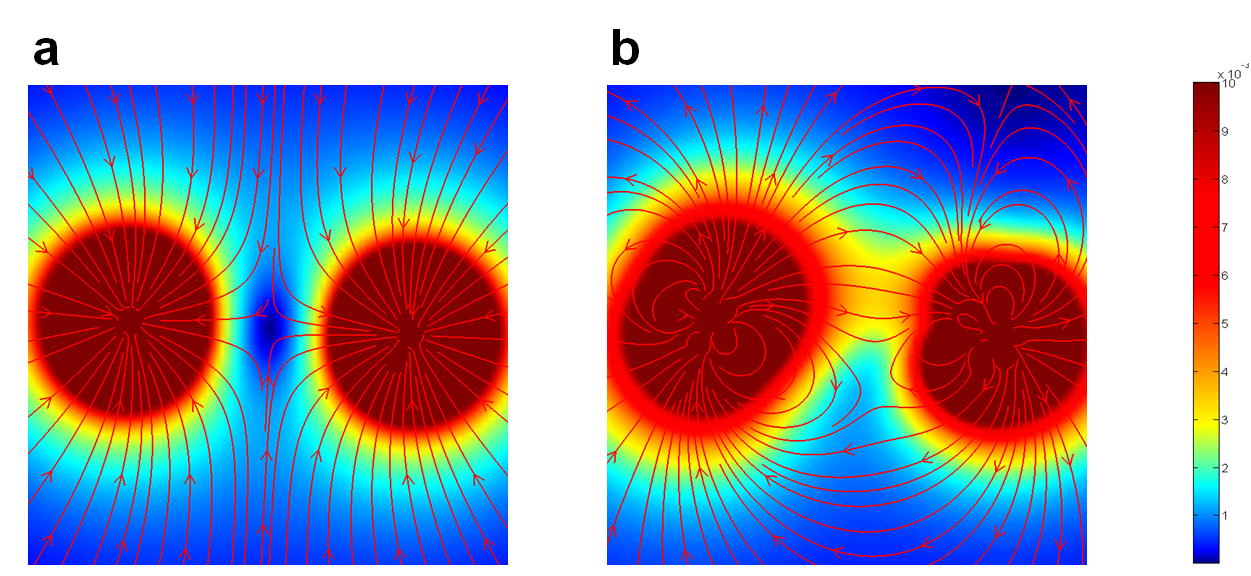
**

Figure S8. Electric field surrounding single macroions with and without mediation of counterions. (a) Two single isolated macroions sitting next to each other. (b) Two single macroions surrounded by counterions, before forming a stable dimer. The arrows show the direction of the field lines and the colors manifest the strength of the electric field.

**
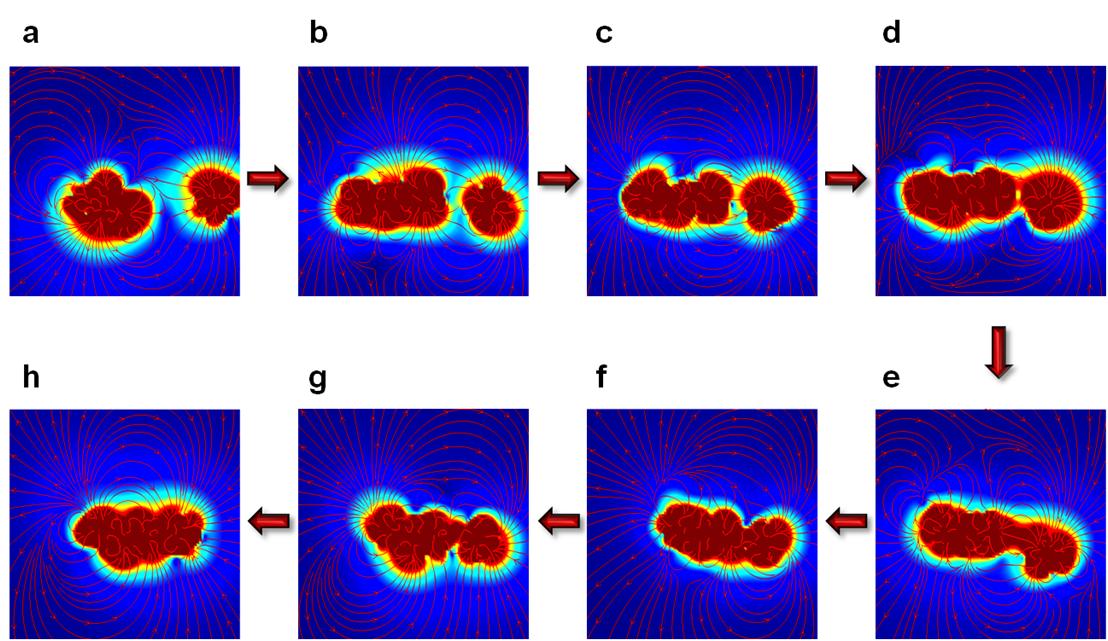
**

Figure S9. Time evolution of the electric field lines and their magnitude surrounding macroions self-assembled into a monolayer with a single macroion merging into it. (a to h) A single macroion merges with a monolayer (assembled by 10 macroions), from the beginning (a) to the end (h). The strength of the electric field is displayed in different colors, from blue to red, weak to strong.

References

(1) Liu, Z.; Liu, T.; Tsige, M. *Sci. Rep.* **2016**, *6*, 26595

(2) Marrink, S. J.; Risselada, H. J.; Yefimov, S.; Tieleman, D. P.; de Vries, A. H. *J. Phys. Chem. B* **2007**, *111*, 7812.

(3) Plimpton, S. *J. Comput. Phys.* **1995**, *117*, 1.

(4) Hockney, R. W.; Eastwood, J. W. *Computer simulation using particles*; Special student ed.; A. Hilger: Bristol England ; Philadelphia, 1988.
